# Motivating Mothers to Recommend Their 20-Year-Old Daughters Receive Cervical Cancer Screening: A Randomized Study

**DOI:** 10.2188/jea.JE20160155

**Published:** 2018-03-05

**Authors:** Tomomi Egawa-Takata, Yutaka Ueda, Akiko Morimoto, Yusuke Tanaka, Asami Yagi, Yoshito Terai, Masahide Ohmichi, Tomoyuki Ichimura, Toshiyuki Sumi, Hiromi Murata, Hidetaka Okada, Hidekatsu Nakai, Masaki Mandai, Shinya Matsuzaki, Eiji Kobayashi, Kiyoshi Yoshino, Tadashi Kimura, Junko Saito, Yumiko Hori, Eiichi Morii, Tomio Nakayama, Mikiko Asai-Sato, Etsuko Miyagi, Masayuki Sekine, Takayuki Enomoto, Yorihiko Horikoshi, Tetsu Takagi, Kentaro Shimura

**Affiliations:** 1 OCEAN Study Group (Obstetrical Gynecological Society of Osaka), Osaka, Japan; 2 Department of Obstetrics and Gynecology, Osaka University Graduate School of Medicine, Osaka, Japan; 3 Department of Obstetrics and Gynecology, Osaka Medical College Graduate School of Medical Sciences, Osaka, Japan; 4 Department of Obstetrics and Gynecology, Osaka City University Graduate School and Faculty of Medicine, Osaka, Japan; 5 Department of Obstetrics and Gynecology, Kansai Medical University Graduate School of Medicine, Osaka, Japan; 6 Department of Obstetrics and Gynecology, Kinki University Graduate School of Medical Sciences, Osaka, Japan; 7 Department of Pathology, Osaka University Graduate School of Medicine, Osaka, Japan; 8 Cancer Control Center, Osaka International Cancer Institute, Osaka, Japan; 9 Department of Obstetrics and Gynecology, Yokohama City University Graduate School of Medicine, Yokohama, Kanagawa, Japan; 10 Department of Obstetrics and Gynecology, Niigata University Graduate School of Medical and Dental Sciences, Niigata, Japan

**Keywords:** cervical cancer screening, daughter, leaflet, mother, recommendation

## Abstract

**Background:**

In Japan, the rate of cervical cancer screening is remarkably low, especially among women in their twenties and thirties, when cervical cancer is now increasing dramatically. The aim of this study was to test whether a modified government reminder for 20-year-old women to engage in cervical cancer screening, acting through maternal education and by asking for a maternal recommendation to the daughter to receive the screening, could increase their participation rate.

**Methods:**

In two Japanese cities, 20-year-old girls who had not received their first cervical cancer screening before October of fiscal year 2014 were randomized into two study arms. One group of 1,274 received only a personalized daughter-directed reminder leaflet for cervical cancer screening. In the second group of 1,274, the daughters and their mothers received a combination package containing the same reminder leaflet as did the first group, plus an additional informational leaflet for the mother, which requested that the mother recommend that her daughter undergo cervical cancer screening. The subsequent post-reminder screening rates of these two study arms were compared.

**Results:**

The cervical cancer screening rate of 20-year-old women whose mothers received the information leaflet was significantly higher than that for women who received only a leaflet for themselves (11% vs 9%, *P* = 0.0049).

**Conclusions:**

An intervention with mothers, by sending them a cervical cancer information leaflet with a request that they recommend that their daughter receive cervical cancer screening, significantly improved their daughters’ screening rate.

## INTRODUCTION

Worldwide, cervical cancer is the fourth most common cancer in women and the fourth leading cause of death in women from cancer.^1^ In many westernized countries, it has been proven that increased screening reduces cervical cancer occurrence and mortality.^2^ However, here in Japan, the cervical cancer screening rate is relatively low compared to other countries of a similar economic level.

In Japan, screening for cervical cancer is recommended to begin at age 20. When a woman approaches this age, her first invitation for screening, along with a free coupon, is sent from her local government between May and July. In addition, every 2 years thereafter, the local government usually gives all women older than age 20 some form of financial aid for cervical screening, so that the cost is either free or only several hundred yen.

In spite of this free or greatly reduced cost, the screening rate for cervical cancer among Japanese women was recently a dismally low 10.2% for 20–25-year-olds, with a somewhat better rate of 24.2% in 26–30-year-olds.^3^ For contrast, in some westernized countries the screening rate is as high as 80% for these same age groups.^2^ As a result of this low screening rate, but also because of changing lifestyles, the incidence of cervical cancer in Japan has recently begun increasing significantly for women in their twenties and thirties.^4^ To reverse this resurgence of cervical cancer incidence and mortality in Japan, it is imperative that we begin improving the cervical cancer screening rate among our youth.

Our previous studies have revealed that a mother’s attitudes towards cancer screening in general, cervical cancer, and anti-cancer vaccinations are closely correlated with her daughters’ cervical cancer screening and vaccination rates.^5^^,^^6^ Our survey interviews have revealed that teen girls, when they approach 20, usually ask their mothers for advice about cervical cancer screening.

To find out whether a modified reminder for 20-year-old women to engage in cervical cancer screening, through maternal education and asking for the mother’s recommendation to the daughter to receive the screening, can increase the daughter’s cervical cancer screening rate, we conducted a randomized study in two cities in Osaka Prefecture, Japan in 2015.

## METHODS

We asked the local governments of two Osaka Prefecture cities, Toyonaka City (population 400,000) and Yao City (270,000), to collaborate with us. These cities usually send their 20-year-old females an invitation and a free coupon for cervical cancer screening between May and July, then in November–December they send reminder letters to any women who had not by then taken advantage of the free screening. As a result of insights obtained from preliminary interviews conducted with several 20-year-old females and their mothers, we revised the standard cervical cancer reminder leaflet for 20-year-olds, and then added a totally new leaflet made just for their mothers.

**Figure 1.**
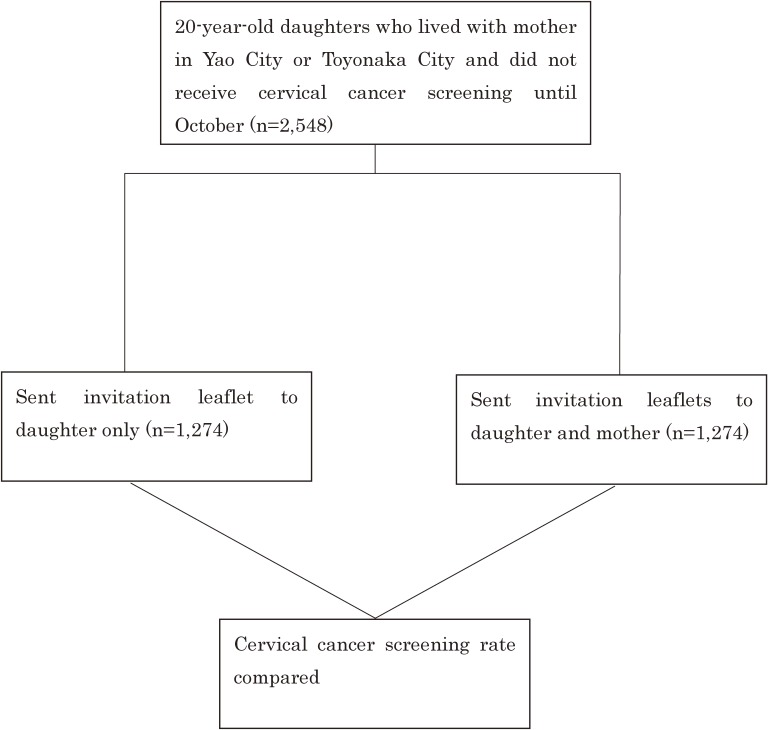
Study design

The 20-year-old females who had not yet received a cervical cancer screening by October and who still lived with their mother were listed in the order of their Japanese syllabary and then randomized into two study groups. In November and December, the informational leaflet meant for the 20-year-old daughter was sent to one group only. In the other group, both the daughter leaflet and the mother leaflet were sent together in one envelope (Figure 1).

**Figure 2.**
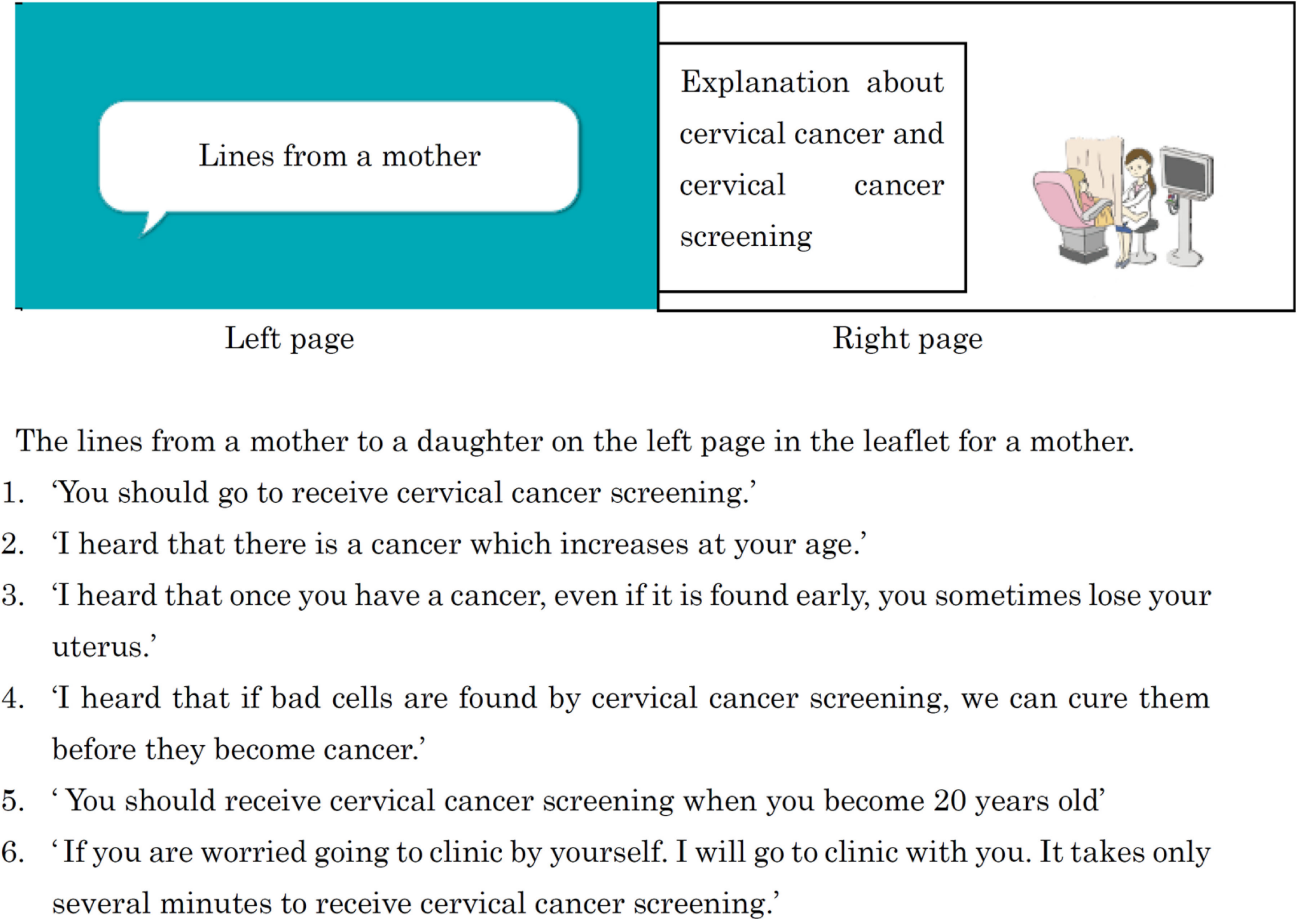
Design of mother leaflet. A mother’s possible conversation with her daughter is on the left page, and an explanation of cervical cancer and cervical cancer screening, with a figure, is on the right page.

The leaflet for the daughter consisted of an informational section, which discussed cervical cancer and the importance of getting screening for cervical cancer, along with a cartoon strip of a conversation between a daughter and her mother (eFigure 1). The left page of the leaflet for the mother consisted of suggestions for simple words which she could use to talk to her daughter about cervical cancer and cervical cancer screening. On the right page were more detailed explanations of cervical cancer and cervical cancer screening. The leaflet implored the mother to recommend to her daughter that she receive cervical cancer screening (Figure 2). Finally, we followed these two groups until the end of March 2015, when the resulting rates of cervical cancer screening were collected and compared. For comparison, we used data obtained from a separate study we had conducted in Hirakata City during the previous fiscal year.^7^

### Statistics

We used Fisher’s exact test and the Chi-square test for statistical analysis. The level of statistical significance was set at *P* = 0.05.

### Ethical consideration

The Osaka University Hospital Medical Ethics Committee approved this research.

### Financial support

A Health and Labor Research Grant (H26-innovative-cancer-research-general-102) and a Japan Agency for Medical Research and Development Grant (15ck0106103h0102) supported this research.

## RESULTS

In December 2014, there were 2,548 females living in the two cities who met our study criteria: being 20 years old, living with their mother, and not having received cervical cancer screening as of October, 2014. We sent only the reminder leaflet meant for the daughter to 1,274 of these females. The other 1,274 were sent both a revised reminder leaflet for the daughter and an informational leaflet for the mother (Figure 1). In the group for which only a leaflet for the daughter was sent, the number of girls who received cervical cancer screening by March of 2015 was 9% (115/1,274), whereas it was significantly higher (11% [146/1,274]; *P* = 0.0049, Table 1), in the group for which the intervention actively involved the mothers.

**Table 1.** Cervical cancer screening rates of the two randomized study groups

Sent a leaflet	Received screening	Not receiving screening	*P* =
Only to daughter	115 (9%)	1,159 (91%)	0.0049
To daughter and mother	146 (11%)	1,128 (89%)	

The total number of eligible 20-year-old females in the two cities was 3,112; 2,548 lived with their mother (and were part of this study). Of the total 3,112 eligible, 386 received cervical cancer screening sometime during fiscal year 2015. Thus, the overall rate of cervical cancer screening was 12% among the total eligible 20-year-olds (Table 2).

**Table 2.** Cervical cancer screening rate of all 20-year-old females

Total number of 20-year-old females	Received screening	Total rate of screening
3,112	386	12%

## DISCUSSION

Following receiving their first invitation and free coupon from May through July for a screening, the initial rate (before October) among 20-year-old females of getting a cervical cancer screening in our two study cities was abysmally low. Thus, any increase in cervical cancer screening for the hold-outs as a result of a more effective November reminder letter would be of great significance. There are several reports that educational leaflets about health promotion in the form of a comic strip are effective with youth.^8^^,^^9^ However, this is the first report of a randomized controlled study showing that positively influencing a mother’s recommendation for cervical cancer screening to her 20-year-old daughter with a comic strip is also an effective means of increasing the rate of screening, moving the dial from 9% to 11% in the study group also receiving the mother’s leaflet. Since the implementation of free coupons for increasing the rate of cervical cancer screening in Japan, this modified reminder leaflet approach has so far proven to be one of the most effective interventions yet undertaken to further improve screening rates.

In comparison, we conducted a parallel study in the Osaka Prefecture city of Hirakata.^7^ During May of fiscal year 2013, only the standard invitation plus free coupon for cervical cancer screening was mailed out by the Hirakata government to all eligible 20-year-old women. As follow-up that year, the standard reminder letters were mailed during January. There was a very small number of women who had already received screening on their own accord in April of 2013, before they received their free coupon in May. Even when we included them, the screening rate over the 8 months prior to the reminder, from April to December, was only 6.4%.

Starting in January of 2014, the standard governmental reminder leaflet was sent to all the remaining eligible 20-year-old women in Hirakata who had not yet been screened by that date. By March of 2014, after the standard reminder, the screening rate for the final 4 months among those who had not been screened by December of 2013 was only 3.6% (the number screened from December 2013 through March of 2014, divided by the residual number who had not been screened prior to the January reminder). We compiled data from these two periods and compared them with results from our study done in fiscal year 2014, using our two revised leaflets.

In May of 2014, the standard invitation and free coupon from the Hirakata government were again sent to all eligible 20-year-old females. However, in contrast to the previous year, in November and December of 2014, our revised daughters’ and mothers’ reminder leaflets were sent out to all women who had not yet received cervical cancer screening. Everyone got the same combination packet because the government of Hirakata would not allow us to conduct a randomized study where half of the eligible group got different information from the other half.^7^

In May of 2015, for the initial call for cervical cancer screening, the Hirakata government sent our revised daughter leaflet, along with the free coupon, to all their eligible 20-year-old women. The rate of cervical cancer screening that occurred before a reminder was sent out in January was significantly higher than the same time period (from invitation to reminder) during the previous year (data not submitted).

We note that the resulting 2% difference between our two study groups, 9% versus 11%, after adding a letter to the mother, was not as dramatic. Regrettably, we could not include a study arm using only the older standard reminder letter for contrast to the revised reminder.

The correlation between a daughter’s cancer screening and her mothers’ knowledge of health-related information is poorly reported in Japan because a person over 20 is regarded as an independent adult. However, by interviewing younger girls, we found that most teenage girls in Japan think that, when they become 20, they will ask for their mother’s opinion about cervical cancer screening. This bodes well, along with the findings we previously reported that the level of a mother’s own cancer health knowledge and cancer screening consultation behavior correlates well with whether or not they had, or would, recommend to their daughters that they should receive cervical cancer screening.^4^

It is important to note that Japan is somewhat exceptional, in that 74% of its 18–24-year-old females are still living with their mothers, whereas only 39% and 44% do so in the United States and United Kingdom, respectively.^10^ A mother’s influence is far greater when the daughter is still living with her. This manner of a mother’s influence over cervical cancer screening is also expected to be effective in other countries where ‘young’ daughters commonly live with their mothers. As a bonus, an informational leaflet directed at the mother may better educate her, improving the mother’s own cervical cancer screening rate. We are now planning to examine the cancer screening rate of the mothers who received our leaflet.

One limitation of this study is that certain participant background factors, such as the socio-economic status among the randomized groups, could not be taken into account because Toyonaka City and Yao City do not permit revelation of this type of personal information.

The proportion of females who had prior sexual experience was also unknown, and such data would have had a large degree of uncertainty anyway because it would require self-reporting, which is suspect. In Japan, roughly 60% of 20-year-old women are reported to have had sexual intercourse experience.^11^^,^^12^ Cervical cancer screening is applicable for women who have not had penetrating sexual intercourse experience because HPV is also transmitted by oral sexual encounters, touching a partner’s genitals, or being touched on the genitals by a partner in ways that many respondents might not consider or report as ‘intercourse’.^13^^,^^14^ However, there are few cases of cervical cancers that are not related to HPV. Therefore, because there is no means to know for sure who has and has not been exposed to HPV, and because the risks from cervical cancer are so high, local governments proactively send a free coupon for cervical cancer screening to all 20-year-old females.

A second limitation of this study is that our approach will be most effective for daughters who still live with their mother; it remains to be seen how effective this will be for daughters and mothers who live separately. In addition, it is not obvious yet how the mothers are communicating their pro-screening recommendations to their daughters. Recently, a follow-up questionnaire about how they used the leaflets was sent to the mothers and daughters. The results of the questionnaire are now under analysis.

Although our combined daughter and maternal education approach has been somewhat effective, we still have to greatly improve the cervical cancer screening rate in Japan (to as high as 90%) if we are ever going to significantly reduce our alarming and ever increasing incidence of cervical cancer. One approach would be to institute an enhanced health education program in junior high school about cervical cancer and cancer screening. Another approach would be to establish an improved registry and invitation/reminder system throughout Japan, even though such efforts may have been conducted by some local governments on a limited basis already.

In conclusion, we have found that an interventional education reminder leaflet for cervical cancer screening sent to the mothers of age-eligible girls, combined with a request that the mothers recommend to their daughters that they receive a free cervical cancer screening, significantly improved their daughters’ cervical cancer screening rate. However, to improve cervical cancer screening to the point that it will significantly impact the incidence of cervical cancer in Japan will require major efforts in public education, beginning in junior high school. Other methods will need to be investigated as well.

## APPENDIX A. SUPPLEMENTARY DATA

eFigure 1. The daughter leaflet. The importance of cervical cancer screening is explained, along with a cartoon depicting a mother/daughter conversation.
