# Supplementary material for: Motivating Mothers to Recommend Their 20-Year-Old Daughters Receive Cervical Cancer Screening: A Randomized Study
Source: J Epidemiol. 2018 Mar 5;28(3):156–60. doi: 10.2188/jea.JE20160155 (PMC5821693; doi:10.2188/jea.JE20160155)
Supplement: Supplementary file 1 [file je-28-156-s001.pdf]

eFigure 1.

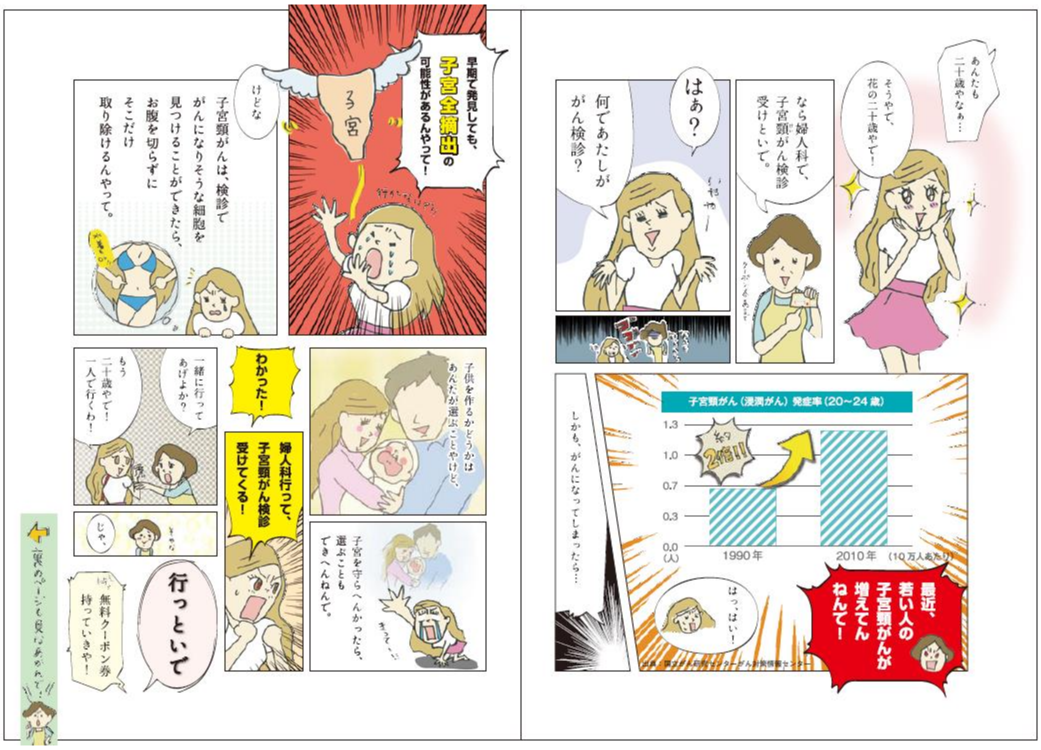

The contents of the cartoon

The girl becomes 20 years old and she says she doesn’t need cervical cancer screening because she is young. Then the mother tells her that cervical cancer in the twenties in Japan is increasing two-fold, and if cervical cancer is found, the uterus should be removed, even if the cancer is early stage. The mother tells her that she should go to receive cervical cancer screening to keep the possibility of having a baby. The daughter is convinced to go to a gynecologic clinic.
